# Non-thermodynamic factors affect competition between thermophilic chemolithoautotrophs from deep-sea hydrothermal vents

**DOI:** 10.1128/aem.00292-24

**Published:** 2024-07-16

**Authors:** Briana C. Kubik, James F. Holden

**Affiliations:** 1 Department of Microbiology, University of Massachusetts, Amherst, Massachusetts, USA; University of Wisconsin-Madison, Madison, Wisconsin, USA

**Keywords:** hydrothermal vents, thermophiles, methanogenesis, sulfur reduction, competition, anaerobes

## Abstract

Various environmental factors, including H_2_ availability, metabolic tradeoffs, optimal growth temperature, stochasticity, and hydrology, were examined to determine if they affect microbial competition between three autotrophic thermophiles. The thiosulfate reducer *Desulfurobacterium thermolithotrophum* (*T*_opt_72°C) was grown in mono- and coculture separately with the methanogens *Methanocaldococcus jannaschii* (*T*_opt_82°C) at 72°C and *Methanothermococcus thermolithotrophicu*s (*T*_opt_65°C) at 65°C at high and low H_2_ concentrations. Both methanogens showed a metabolic tradeoff shifting from high growth rate–low cell yield at high H_2_ concentrations to low growth rate–high cell yield at low H_2_ concentrations and when grown in coculture with the thiosulfate reducer. In 1:1 initial ratios, *D. thermolithotrophum* outcompeted both methanogens at high and low H_2_, no H_2_S was detected on low H_2_, and it grew with only CO_2_ as the electron acceptor indicating a similar metabolic tradeoff with low H_2_. When the initial methanogen-to-thiosulfate reducer ratio varied from 1:1 to 10^4^:1 with high H_2_, *D. thermolithotrophum* always outcompeted *M. jannaschii* at 72°C. However, *M. thermolithotrophicus* outcompeted *D. thermolithotrophum* at 65°C when the ratio was 10^3^:1. A reactive transport model that mixed pure hydrothermal fluid with cold seawater showed that hyperthermophilic methanogens dominated in systems where the residence time of the mixed fluid above 72°C was sufficiently high. With shorter residence times, thermophilic thiosulfate reducers dominated. If residence times increased with decreasing fluid temperature along the flow path, then thermophilic methanogens could dominate. Thermophilic methanogen dominance spread to previously thiosulfate-reducer-dominated conditions if the initial ratio of thermophilic methanogen-to-thiosulfate reducer increased.

**IMPORTANCE:**

The deep subsurface is the largest reservoir of microbial biomass on Earth and serves as an analog for life on the early Earth and extraterrestrial environments. Methanogenesis and sulfur reduction are among the more common chemolithoautotrophic metabolisms found in hot anoxic hydrothermal vent environments. Competition between H2-oxidizing sulfur reducers and methanogens is primarily driven by the thermodynamic favorability of redox reactions with the former outcompeting methanogens. This study demonstrated that competition between the hydrothermal vent chemolithoautotrophs *Methanocaldococcus jannaschii*, *Methanothermococcus thermolithotrophicus*, and *Desulfurobacterium thermolithotrophum* is also influenced by other overlapping factors such as staggered optimal growth temperatures, stochasticity, and hydrology. By modeling all aspects of microbial competition coupled with field data, a better understanding is gained on how methanogens can outcompete thiosulfate reducers in hot anoxic environments and how the deep subsurface contributes to biogeochemical cycling.

## INTRODUCTION

The rocky subseafloor harbors an estimated 40% of the overall bacterial and archaeal biomass on Earth (1, 2), but the local and global biogeochemical impact of this life is largely unknown. Deep-sea hydrothermal vents provide a window into the fundamental microbial and biogeochemical processes that occur within the rocky subseafloor. At Axial Seamount, the chemistries of low-temperature (<50°C) hydrothermal fluids from two sites, Marker 113 and Marker 33, had similar pH and concentrations of H_2_S, ΣCO_2_, and CH_4_ suggesting overall similarity in the source fluids (3, 4) (Table S1). However, among the thermophiles collected from exiting low-temperature hydrothermal fluid, Marker 113 was mostly populated by the methanogens *Methanocaldococcus* and *Methanothermococcus*, while Marker 33 was mostly the sulfur and thiosulfate reducer *Desulfurobacterium* based on metagenomic and culture-based analyses (4, 5) (Fig. S1). This suggests that these thermophilic, hydrogenotrophic autotrophs at these sites occupy similar ecological niches and likely compete for H_2_ but with differing outcomes. The purpose of this study was to examine possible factors that lead to one group of organisms outcompeting the other in hydrothermal systems.

Models are used to predict microbial competition and biogeochemical impacts using a combination of deterministic and stochastic variables. Competition between chemolithoautotrophs at hydrothermal vents is often predicted using thermodynamic models based on the Gibbs energy available for given metabolisms and the fluid geochemistry of the environment (6–8). However, in resource-limited environments, microorganisms can undergo a metabolic tradeoff between microbial growth rate and cell yield (*Y*_*x*/*p*_, biomass produced/mole of metabolite produced) (9, 10), which may provide a competitive advantage for some organisms. Previous work showed that *Methanocaldococcus jannaschii* switched from fast growth rates and low growth yields in high H_2_ conditions to slow growth rates and high growth yields in low H_2_ conditions (11). However, it is unknown if a similar tradeoff occurs in thermophilic autotrophic sulfur/thiosulfate reducers and how this might impact competition in resource-limited environments. Also, staggered optimal growth temperatures of thermophilic organisms can create competitive advantages depending on subseafloor geometry and hydrology, which can vary the residence time of hydrothermal fluids at different temperatures (12). Furthermore, stochastic factors, such as dispersal rates, drift, and population bottlenecks, can create niche exclusion and affect community composition (13). Some vent sites experience large shifts in microbial community composition following volcanic eruptions (14–16) possibly triggering stochastic recolonization events of new vent sites that could impact the outcome of competition.

In this study, competition between the thermophilic anaerobic chemolithoautotrophs *Methanocaldococcus*, *Methanothermococcus*, and *Desulfurobacterium* was examined to determine if the predominance of a type of organism or its metabolism was due primarily to the Gibbs energy for its metabolism or under what circumstances other factors, such as H_2_ availability, metabolic tradeoffs, relative cell concentration, incubation temperature, and hydrology, might influence the outcome of competition. These data were used to parameterize a non-dimensional reactive transport model that included thermophilic and hyperthermophilic hydrogenotrophic methanogenesis and thermophilic hydrogenotrophic thiosulfate reduction and considered varying parameters, such as initial cell concentrations and residence times, of the fluids at different temperatures.

## RESULTS

### H_2_ limitation and metabolic tradeoffs

**Fig 1:**
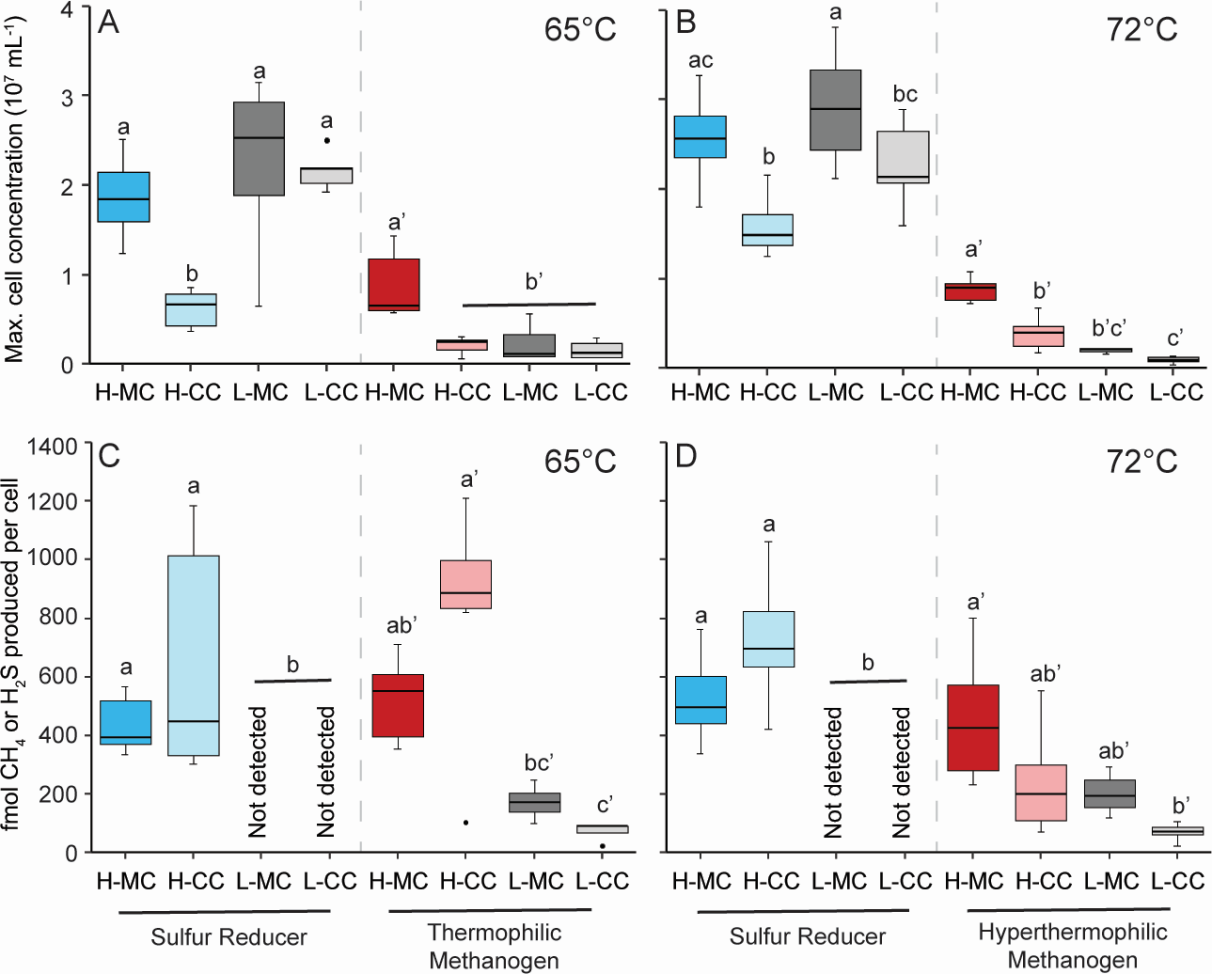
Growth of *D. thermolithotrophum* and *M. thermolithotrophicus* at 65°C (**A, C**) and *D. thermolithotrophum* and *M. jannaschii* at 72°C (**B, D**). The maximum cell concentrations (**A, B**) and the growth product yields (**C, D**) are shown. *D. thermolithotrophum* at high initial H_2_ concentration is shown in blue shades, methanogens at high initial H_2_ concentration are shown in red shades, and *D. thermolithotrophum* and methanogens at low initial H_2_ concentrations are both shown in gray shades. Monocultures are the darker shades of all colors, while cocultures are the lighter shades. The statistical relevance (*P* < 0.05) of the data is shown separately for each organism (non-prime versus prime). Abbreviations: MC, monoculture; CC, coculture in 1:1 initial cell ratio; H, high initial H_2_ concentration; L, low initial H_2_ concentration.

H_2_ availability for cell growth was assumed to become increasingly limited going from monoculture to coculture conditions and from high initial H_2_ in the headspace to low initial H_2_ (left-to-right for each organism in Fig. 1). Each set of high and low H_2_ incubations ended when *Desulfurobacterium thermolithotrophum* in monoculture reached late logarithmic growth phase (~4–5 cell doublings). For each condition, *D. thermolithotrophum* grew to a higher cell concentration than *Methanothermococcus thermolithotrophicus* at 65°C (Fig. 1A) and *M. jannaschii* at 72°C (Fig. 1B). At both growth temperatures, the maximum cell concentrations of *D. thermolithotrophum* were generally the same for high H_2_ monocultures, low H_2_ monocultures, and low H_2_ cocultures but lower for high H_2_ cocultures (Fig. 1A and B). Similarly, the specific growth rates of *D. thermolithotrophum* were the same for all four growth conditions at 65°C and in five of six pairwise comparisons at 72°C (Fig. S2A and B).

At both growth temperatures, the maximum cell concentration of the methanogens was lower in high H_2_ coculture, low H_2_ monoculture, and low H_2_ coculture conditions relative to high H_2_ monoculture conditions (Fig. 1A and B). At 65°C, the specific growth rate of *M. thermolithotrophicus* was unchanged in coculture relative to monoculture at both high and low H_2_ concentrations but was lower at low H_2_ concentrations relative to high H_2_ concentrations (Fig. S2A and B). At 72°C, the specific growth rate of *M. jannaschii* was lower in coculture relative to monoculture at both high and low H_2_ concentrations.

At both temperatures, each methanogen decreased the amount of CH_4_ produced per cell in low H_2_ coculture conditions relative to high H_2_ monoculture conditions (Fig. 1C and D). As a result, the cell yield increased from 1.9 × 10^12^ cells/mol CH_4_ produced by both high H_2_ monocultures to 1.4 × 10^13^ cells/mol CH_4_ produced by both low H_2_ co-cultures indicating a rate–yield metabolic tradeoff with decreasing H_2_ availability. In high H_2_ conditions, *D. thermolithotrophum* produced 577 fmol H_2_S/cell, and cell yield was 1.7 × 10^12^ cells/mol H_2_S produced at both temperatures in mono- and coculture (Fig. 1C and D). However, no H_2_S was detected when *D. thermolithotrophum* was grown in low H_2_ conditions despite having the same maximum cell concentrations and specific growth rates as in high H_2_ conditions (Fig. 1C and D; Fig. S2A and B). This suggests that *D. thermolithotrophum* may not require thiosulfate as a terminal electron acceptor, especially at low H_2_ concentrations, and increased its cell yield on low H_2_ also as part of a rate–yield metabolic tradeoff.

**Fig 2:**
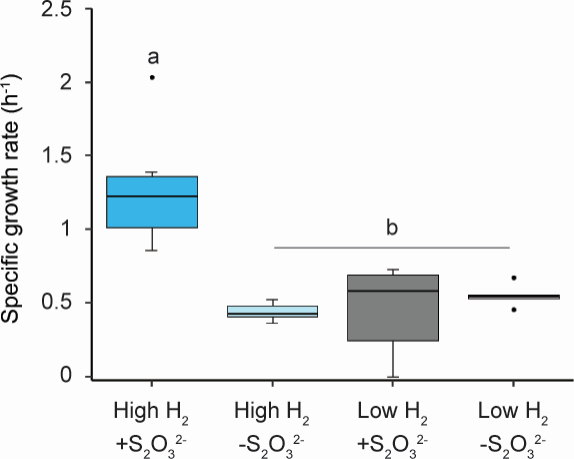
Specific growth rate of *D. thermolithotrophum* in monoculture at 72°C at high (blue shade) and low (gray shade) initial H_2_ concentrations with and without the addition of sodium thiosulfate as a terminal electron acceptor. The statistical relevance of the data is *P* < 0.05.

To test this, *D. thermolithotrophum* was grown at high and low H_2_ concentrations without the addition of Na_2_S_2_O_3_ or any other terminal electron acceptor other than CO_2_. At high H_2_ concentration, the specific growth rate of *D. thermolithotrophum* with thiosulfate was 1.28 ± 0.19 h^−1^ (95% CI) (Fig. 2). With high H_2_ but without thiosulfate, *D. thermolithotrophum* grew at a specific growth rate of 0.44 ±0.07 h^−1^. At low H_2_ concentration, the specific growth rates of *D. thermolithotrophum* with and without thiosulfate were each the same as for high H_2_ concentration without thiosulfate (Fig. 2).

### Competition at varying initial methanogen:thiosulfate reducer ratios

**Fig 3:**
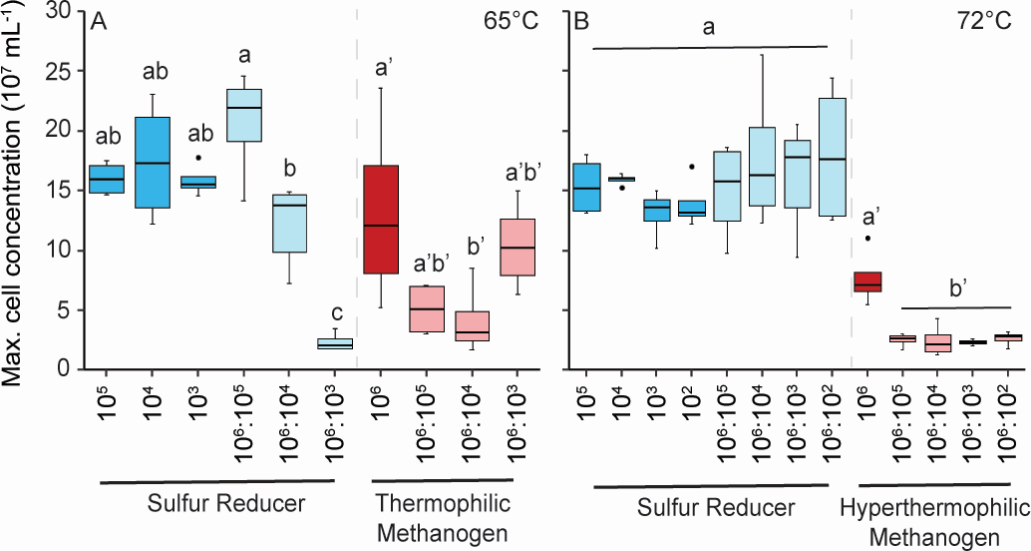
Maximum cell concentrations of *D. thermolithotrophum* and *M. thermolithotrophicus* at 65°C (**A**) and *D. thermolithotrophum* and *M. jannaschii* at 72°C (**B**). *D. thermolithotrophum* at high initial H_2_ concentration is shown in blue shades, methanogens at high initial H_2_ concentration are shown in red shades, and *D. thermolithotrophum* and methanogens at low initial H_2_ concentrations are both shown in gray shades. Monocultures are the darker shades of all colors, while cocultures are the lighter shades. The statistical relevance (*P* < 0.05) of the data is shown separately for each organism (non-prime versus prime). The initial cell concentrations and initial ratios of methanogens to *D. thermolithotrophum* cells is shown along the *x*-axis for each condition.

Each incubation ended separately when the cultures for each condition reached stationary growth phase. When *D. thermolithotrophum* was grown at 65°C in monoculture and in coculture with *M. thermolithotrophicus*, the maximum cell concentrations of *D. thermolithotrophum* at the end of growth were the same for all monoculture conditions and for 10^6^:10^5^ and 10^6^:10^4^ initial ratios of *M. thermolithotrophicus:D. thermolithotrophum* (Fig. 3A). However, the maximum cell concentration of *D. thermolithotrophum* at the end of growth decreased significantly when the initial cell ratio was 10^6^:10^3^ of *M. thermolithotrophicus:D. thermolithotrophum* relative to the monocultures and for coculture ratios of 10^6^:10^4^ relative to 10^6^:10^5^. The maximum cell concentration of *M. thermolithotrophicus* at the end of growth for cells grown at 65°C and 10^6^:10^4^ of *M. thermolithotrophicus:D. thermolithotrophum* was lower than that of *M. thermolithotrophicus* grown in monoculture, but the maximum cell concentration at the end of growth for the methanogen at 10^6^:10^3^ initial cell ratio was the same as the monoculture (Fig. 3A).

At 72°C, the maximum cell concentrations of *D. thermolithotrophum* at the end of growth were the same for all mono- and *M. jannaschii* coculture conditions regardless of their initial cell concentration (Fig. 3B). The maximum cell concentration of *M. jannaschii* at the end of growth in coculture with *D. thermolithotrophum* was lower than that of *M. jannaschii* in monoculture for all initial cell ratios (Fig. 3B).

### Reactive transport modeling

**Fig 4:**
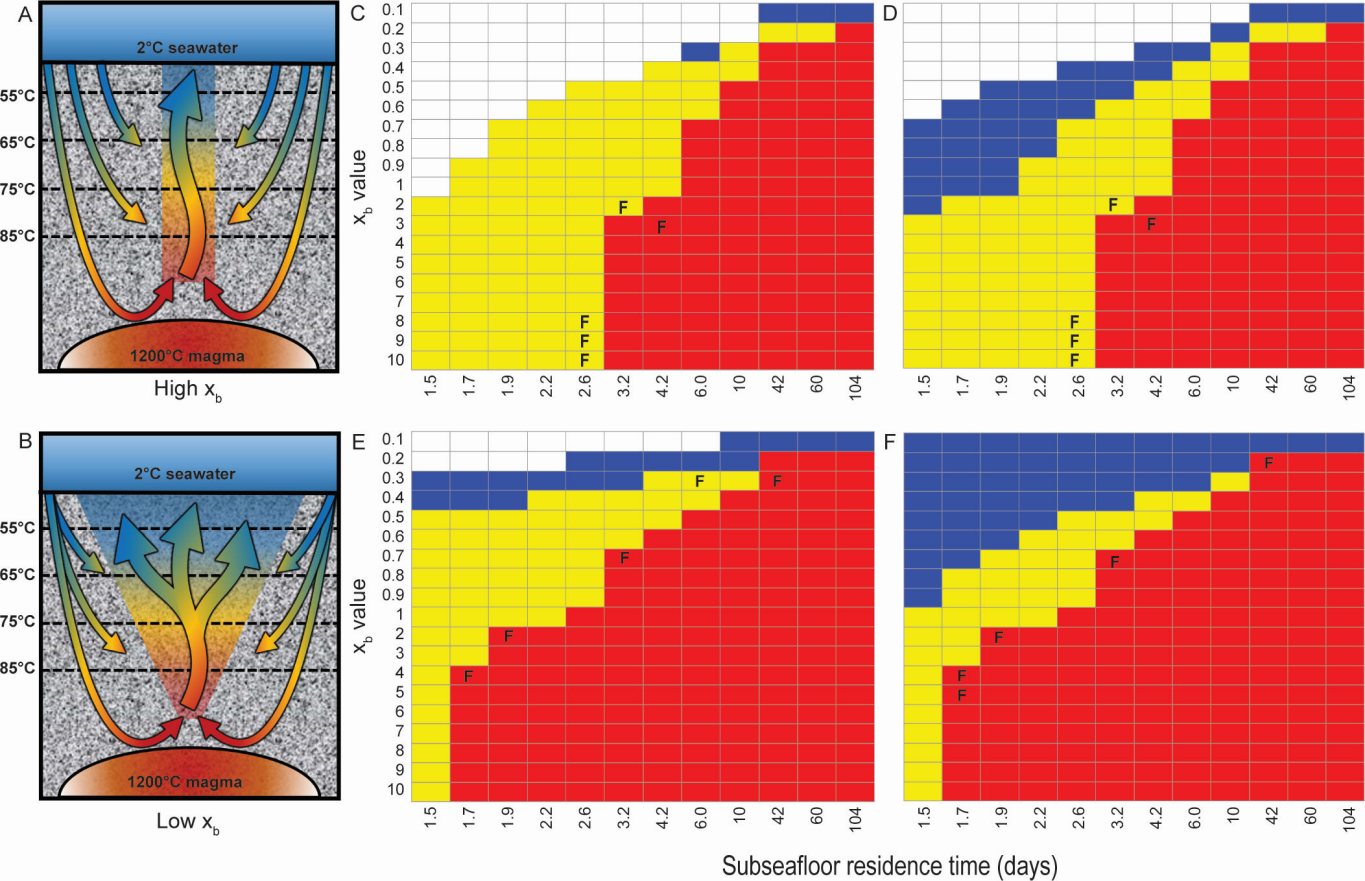
Reactive transport modeling results for (**A**) pipe-like (high *x*_*b*_) and (**B**) plume-like (low *x*_*b*_) fluid flow. The heat maps (**C–F**) show the predominant thermophile (*M. jannaschii* in red, *D. thermolithotrophum* in yellow, and *M. thermolithotrophicus* in blue) at varying *x*_*b*_ values and subseafloor residence times below 85°C. H_2_ concentrations in and temperature of pure hydrothermal vent fluids were 300 µM and 214°C (**C, D**) and 950 µM and 330°C (**E, F**) representing Marker 33 and Marker 113, respectively, at Axial Seamount. The initial *M. jannaschii:D. thermolithotrophum:M. thermolithotrophicus* ratios were either 1:1:1 (**C, E**) or 1:1:100 (**D, F**). The conditions that best fit the field results for Marker 33 and Marker 113 are indicated with an F symbol in the heat map.

The outcome of competition between hyperthermophilic and thermophilic methanogens and thermophilic autotrophic thiosulfate reducers was predicted using a reactive transport model with varying hydrologic shape functions (*x*_*b*_) and dilution rates (*Q*′_vt_) at varying temperatures (Fig. 4). High *x*_*b*_ values represent pipe-like flow with little variation in residence time at each temperature as fluid flows through the model and temperature decreases (Fig. 4A), while low *x*_*b*_ values represent plume-like flow with increasing residence time (1/*Q′*_vt_) as fluid flows through the model and temperature decreases (Fig. 4B). For all conditions, hyperthermophilic methanogens were dominant at low *Q′*_vt_ values (i.e., high residence times) (Fig. 4C through F). Thermophilic autotrophic thiosulfate reducers were dominant when *Q′*_vt_ values increased (i.e., relatively lower residence times) and when *x*_*b*_ values decreased (i.e., residence times increase with decreasing temperature). Thermophilic methanogens similarly were dominant as *Q′*_vt_ values continued to increase and *x*_*b*_ values decreased.

When initial thermophilic methanogen cell concentrations were increased to 10^2^-fold higher than the hyperthermophilic methanogens and thermophilic autotrophic thiosulfate reducers, the thermophilic methanogens were increasingly dominant at high *Q′*_vt_–low *x*_*b*_ values (Fig. 4D and F) relative to equal initial cell concentrations for the three organisms (Fig. 4C and E). Thermophilic methanogens were also more dominant when H_2_ concentrations in pure hydrothermal vent fluid increased from 300 µM (Fig. 4C and D) to 950 µM (Fig. 4E and F). When compared with cell proportions and CH_4_ anomalies measured in hydrothermal fluids from Marker 33 and Marker 113 at Axial Seamount (4, 5), the modeling results suggest that the hydrothermal fluid residence times from 85°C to exiting the seafloor at these sites are 63–100 and 40–77 h, respectively (Fig. 4C through F). Marker 33 showed more pipe-like fluid flow, while Marker 113 showed more plume-like fluid flow.

## DISCUSSION

Understanding the factors that influence competition between thermophilic chemolithoautotrophs in hydrothermal environments provides insight into modern subseafloor processes as well as life on the early Earth and the search for extraterrestrial life. In this study, the thermophilic autotrophic thiosulfate reducer *D. thermolithotrophum* outcompeted thermophilic and hyperthermophilic methanogens at high and low H_2_ concentrations when initially in equal cell concentrations with abundant thiosulfate present in the microcosms. This result was expected based on the standard Gibbs energy for each reaction with thiosulfate reduction producing more energy for growth than methanogenesis (8). However, differences in optimal growth temperatures and temperature growth ranges for the three groups of thermophiles influenced their predicted competition in a reactive transport model. Because hydrothermal fluid is the source of H_2_, hyperthermophilic methanogens have sole access to H_2_ among the three groups of organisms until the hydrothermal fluid–seawater mixture cools to temperatures in the range of the other two groups. Hyperthermophilic methanogens dominated when residence time was sufficiently high between 85°C and 72°C. Both microcosm experiments and the reactive transport model showed that thermophilic methanogens had an increasing predicted competitive advantage when the residence time of the fluid increased with decreasing temperature (i.e., low *x*_*b*_ or plume-shaped geometry) and when they initially outnumber thermophilic thiosulfate reducers at least 10^2^- to 10^3^-fold.

H_2_ availability is important for the ability of methanogens to compete with autotrophic thiosulfate reducers. Thermophilic methanogens were more predominant in the reactive transport model when the H_2_ concentration in pure hydrothermal vent fluid increased from 300 to 950 µM and when the residence time of fluids increased with decreasing fluid temperatures (plume-shaped geometry). Monod kinetics showed that *M. jannaschii* had an H_2_ *K*_s_ of 67 µM and required at least 17–23 μM H_2_ for growth in a bioreactor (3). For *D. thermolithotrophum* HR11, H_2_ *K*_s_ was 30 µM and required at least 3 µM H_2_ for growth in a bioreactor (17). The maximum growth rate/H_2_*–K*s ratio for *D. thermolithotrophum* HR11 is fourfold higher than that for *M. jannaschii* (17). Therefore, high-temperature methanogens appear to require highly elevated H_2_ concentrations (or fluxes) to compete with *Desulfurobacterium* for H_2_. In nature, high-temperature methanogens are often more abundant than *Aquificales*, including *Desulfurobacterium*, at vent sites with higher H_2_ concentrations (15, 16, 18–20). *Desulfurobacterium* is often more abundant than high-temperature methanogens at vent sites with more moderate H_2_ concentrations (4, 21–23).

The rate–yield metabolic tradeoff represents two divergent strategies that are important for microbial competition and biogeochemistry. The first is slow growth but efficient metabolism and high cell yields when resources are scarce; the second, fast growth but inefficient metabolism and low cell yields upon rich resources (9, 10). This tradeoff was shown for *M. jannaschii*, which decreased its growth and CH_4_ production rates and increased its cell yield when grown with very low H_2_ flux to the cells (11). This might have provided thermophilic methanogens with a growth advantage over *Desulfurobacterium* spp. This study demonstrated that a similar rate–yield metabolic tradeoff occurs in *D. thermolithotrophum* when it is shifted from high to low H_2_ concentrations. It also suggested that *D. thermolithotrophum*, which was believed to be an obligate sulfur or thiosulfate reducer, could use CO_2_ as its sole added terminal electron acceptor through CO_2_ fixation. At high initial H_2_ concentrations, *D. thermolithotrophum* grew faster when thiosulfate was available as a terminal electron acceptor but grew slowly without the addition of thiosulfate. At low initial H_2_ concentrations, the growth rates were the same, and no H_2_S was detected with or without the addition of thiosulfate. Therefore, the organism may only require sulfur or thiosulfate for extra energy generation when the environment has a high flux of H_2_.

*D. thermolithotrophum* BSA fixes CO_2_ using the Arnon–Buchanan cycle (i.e., the reductive TCA cycle) and relies on ATP citrate lyase to split citrate into oxaloacetate and acetyl-CoA for biosynthesis (24, 25). Based on its whole-genome sequence (26), *D. thermolithotrophum* HR11 has a Hyn-type (Group 1) hydrogenase in its membrane with a twin-arginine transport signal suggesting that its catalytic subunit is oriented toward the periplasm. It also encodes all the genes for the Arnon–Buchanan cycle including a membrane-bound fumarate reductase. This suggests that *D. thermolithotrophum* oxidizes H_2_ in the periplasm and passes electrons to menaquinones, which *D. thermolithotrophum* BSA possesses (24), and then to fumarate reductase where the electrons enter the Arnon–Buchanan cycle for CO_2_ reduction. A proton motive force for ATP generation via oxidative phosphorylation is generated by the oxidation of H_2_ and menaquinones by hydrogenase and fumarate reductase, respectively. Using CO_2_ as a terminal electron acceptor as well as for biosynthesis would provide *D. thermolithotrophum* with a growth advantage in hydrothermal environments that can often be limited in exogenous electron acceptors at high growth temperatures.

Subseafloor hydrology was previously shown to impact methanogen community composition at Axial Seamount. The plume-like (low *x*_*b*_) hydrology of Marker 113 resulted in a shorter residence time for hotter fluid compared to cooler fluid, favoring *Methanothermococcus*, while the pipe-like (high *x*_*b*_) geometry of Marker 33 had a longer residence time at hotter temperatures favoring *Methanocaldococcus* (12). In this study, the non-dimensional reactive transport model from Stewart et al. (12) was expanded to include thermophilic autotrophic growth of *Desulfurobacterium* spp., which are also present in this system (4). The new modeling results support the idea that Marker 113 has shorter residence times for hotter fluid, while Marker 33 has a longer residence time for hotter fluid. Using both CH_4_ anomaly in hydrothermal fluids and measured proportions of *Methanocaldococcus*, *Methanothermococcus*, and *Desulfurobacterium* based on metagenomics to determine the best fit, the modeling results suggest that the residence time of the fluids between 85°C and exiting the seafloor increased from what was reported by Stewart et al. (12). This increase in residence time is understandable given the increased demand for H_2_ with the additional non-methanogenic hydrogenotroph. Furthermore, the model showed that stochastic effects, such as *Methanothermococcus*, initially outnumbering *Desulfurobacterium* 10^2^-fold can increase the *Q′*_vt_*−x*_*b*_ parameter space where *Methanothermococcus* can become the predominant thermophile in the system. Therefore, the stochastic colonization and establishment of an organism with relatively lower Gibbs energy for its metabolism may lead to that organism predominating a niche by excluding an organism with a higher Gibbs energy for its metabolism through a combination of relative abundances of organisms and relative residence times at varying temperatures in the system.

In conclusion, this study demonstrated the utility of coupling metagenomics, field geochemistry, thermodynamic predictions, and biogeochemical modeling to formulate hypotheses regarding the distribution and metabolic potential of microbes (27). Thermodynamic predictions of Gibbs energy associated with various autotrophic metabolisms remain a valid predictor of which thermophilic microbes will predominate in hydrothermal environments. In most instances, the thermophilic thiosulfate reducers in this study outcompeted the thermophilic methanogens. However, this study also showed the importance of other factors, such as staggered optimal growth temperatures, hydrology, and stochastic effects, and how in certain circumstances methanogens might outcompete thiosulfate reducers. Future thermodynamic predictions and biogeochemical modeling may also need to account for a shift in metabolism toward increased CO_2_ fixation and less metabolic product formation as a means for autotrophic growth. Other aspects of competition modeling that were not accounted for herein are the formation of biofilms in the subseafloor, possible spatial separation of metabolic processes within those biofilms, and antibiotic production. Pressure likely has a minor impact on microbial competition as little effect was observed on the growth, community composition, and metatranscriptomes of a natural assemblage of thermophilic hydrogenotrophic autotrophs sampled from Marker 33 hydrothermal fluid when incubated at 55°C on the seafloor at *in situ* pressure relative to a parallel shipboard incubation at 0.1 MPa (28).

## MATERIALS AND METHODS

### Growth medium and microorganisms used

Three thermophilic organisms were obtained from the Deutsche Sammlung von Mikroorganismen und Zellkulturen (DSMZ, Braunschweig, Germany) and used for mono- and coculture experiments: *M. jannaschii* DSM 2661 (*T*_opt_ 82°C) (29), *M. thermolithotrophicus* DSM 2095 (*T*_opt_ 65°C) (30), and *D. thermolithotrophum* HR11 (*T*_opt_ 72°C) (DSM 100454) (17, 26). All chemical reagents were purchased from Sigma-Aldrich. The growth medium is based on DSM medium 282 (29) and is composed of the following per liter: 30 g of NaCl, 4.1 g of MgCl_2_∙6H_2_O, 3.40 g of MgSO_4_∙7H_2_O, 0.33 g of KCl, 0.25 g of NH_4_Cl, 0.14 g of CaCl_2_∙2H_2_O, 0.14 g of K_2_HPO_4_, 10 mL of DSM medium 141 trace elements solution, 10 mL of DSM medium 141 vitamins solution, 0.1 mL of 0.1% (wt/vol) each of Na_2_WO_4_∙2H_2_O and Na_2_SeO_4_ solution, 1 g of NaHCO_3_, 1 g of Na_2_S_2_O_3_, and 50 µL of 0.5% (wt/vol) resazarin. The medium was pH balanced to 6.00 ± 0.05. The medium was reduced with 0.025% (wt/vol) cysteine-HCl. All cells were grown in 60-mL serum bottles sealed with butyl rubber stoppers containing 25 mL of growth medium. For high H_2_ conditions, 1.6 atm of H_2_ and 0.4 atm of CO_2_ were added to the headspace of each bottle based on the standard growth medium (29). For low H_2_ conditions, which was based on the minimum amount of H_2_ needed to support monoculture growth of each organism, 0.1 atm of H_2_, 1.5 atm of N_2_, and 0.4 atm of CO_2_ were added to the headspace of each bottle. All gases were provided by AirGas. Using the geochemical modeling program Geochemist’s Workbench 10.0, the estimated aqueous H_2_ concentrations were 1.2 mM and 85 µM for the high and low H_2_ conditions, respectively, and were representative of the expected H_2_ concentrations at Marker 113 and Marker 33 (3, 12).

### Mono- and coculture incubations

To determine how H_2_ concentration affects cell yield and growth competition, growth medium in serum bottles were inoculated separately with *D. thermolithotrophum*, *M. jannaschii*, and *M. thermolithotrophicus* each in monoculture or in coculture between *D. thermolithotrophum* and *M. jannaschii* or *D. thermolithotrophum* and *M. thermolithotrophicus* (Fig. S3). A logarithmic growth phase culture was used to inoculate each bottle such that the initial concentration of each organism in each bottle was 10^6^ cells/mL based on the detection limit of the Petroff–Hausser counting chamber. *D. thermolithotrophum* and *M. jannaschii*, in mono- and coculture, were incubated at 72°C (the optimum growth temperature of *D. thermolithotrophum*), while *D. thermolithotrophum* and *M. thermolithotrophicus*, in mono- and coculture, were incubated at 65°C (the optimum growth temperature of *M. thermolithotrophicus*). Each combination of cells and temperatures was incubated separately using high and low H_2_ concentrations as described above.

The concentration of total cells in each bottle was determined at various time points during growth using light microscopy (Nikon Eclipse 55*i*) and a Petroff–Hausser counting chamber. The proportions of methanogens and *D. thermolithotrophum* in each coculture sample were determined using fluorescence microscopy (Nikon Eclipse 55*i* with a CoolLED pE-300 light source) and the autofluorescence and coccoid shape of the methanogens (30) relative to the non-fluorescent rod-shaped *D. thermolithotrophum* (17) (Fig. S4). All sample bottles were removed from the incubator when one of the organisms in monoculture (usually *D. thermolithotrophum*) reached late logarithmic-to-early stationary growth phase. The specific growth rate (per h) of each organism in mono- and coculture was determined by fitting an exponential curve to cell concentration versus time. NaOH (0.1 M final concentration) was added to each bottle. The amount of CH_4_ in each bottle was determined by measuring the volume of gas in the bottle and using a gas chromatograph fitted with a flame ionization detector (SRI 8610C) and a HayeSep D packed column (Supelco, 6′×⅛″ stainless steel). The amount of sulfide in each bottle was determined using a spectrophotometer (Thermo Spectronic Genesys 10) and methylene blue (31). The product yield per cell was estimated from the total amount of H_2_S or CH_4_ produced per bottle divided by the total number of thiosulfate reducers or methanogens per bottle, respectively. Data are presented in Table S2.

To determine how initial methanogen:thiosulfate reducer cell concentrations (per mL) ranging from 10^6^:10^5^ to 10^6^:10^2^ affect growth competition, growth medium in serum bottles was inoculated as described above except that the initial cell concentration of *D. thermolithotrophum* varied at 10^5^, 10^4^, 10^3^, and 10^2^ cells/mL relative to 10^6^ cells/mL for each methanogen (Fig. S5). All samples were incubated at 72°C or 65°C as described above with the high H_2_ concentration only. Maximum cell concentrations and the total amount of methane and sulfide produced at the end of the incubation were determined as described above. The cells in each growth condition were allowed to reach late logarithmic-to-early stationary growth phase before removal from the incubator. Data are presented in Tables S3 and S4.

A two-way analysis of variance with a Tukey honestly significant difference *post-hoc* analysis was used to compare one dependent variable (maximum cell concentration, growth rate, growth yield) based on two independent variables as well as any potential interaction between the two (H_2_ concentration and mono- versus coculture conditions). Comparisons were not made across temperatures or organisms except when that was the only variable. A two-sample statistical test (either Student’s *t*-test or Wilcoxon rank sum) was used to compare the maximum cell concentrations of the thiosulfate reducer and methanogens in coculture to determine which organisms outcompete the other. All statistical analyses were performed in RStudio using R statistical software.

### Reactive transport model

The reactive transport model used was a modification of that described previously by Stewart et al. (12) with the addition of hydrogenotrophic, thermophilic thiosulfate reduction to the model. The model described the growth of hydrogenotrophic methanogens and thiosulfate reducers in a mixture of pure high-temperature hydrothermal fluid and 2°C seawater with unlimited thiosulfate that was transported along a one-dimensional flow path consisting of a series of *n* boxes. High-temperature hydrothermal fluid, which naturally lacks Mg^2+^, entered the first box with the fluid composition of pure high-temperature hydrothermal fluid, then flowed from box-to-box, and at each box was progressively diluted with an equal amount of 2°C seawater (composition in Table S5) until it exits the last box with a fluid composition like that observed for low-temperature hydrothermal vents on the seafloor. Because Mg^2+^ is biologically conservative, it acts as a tracer for the dilution of hydrothermal fluid with seawater, which contains Mg^2+^, and is used to constrain the reactive transport model with field data. Because the true length of the flow path is unknown, the model was non-dimensionalized with respect to space such that the sum of all the box volumes was equal to one and represents the total volume of the subseafloor mixing zone feeding the vent outflow.

The total residence time of fluid in the system is set by the spatially non-dimensionalized fluid flux exiting the seafloor (*Q′*_vt_), which has units of time^−1^ and can be thought of as a dilution factor that defines the timescale of hydrothermal fluid circulation. The residence time fluid spends at different temperatures along the flow path is controlled by both *Q′*_vt_ and the volume of each individual box along the flow path. To simplify the specification of box volumes, the volume of each box is given by the following formula:


(Eqn. 1) $$\Delta V_{i}=\frac{1}{n}\cdot \frac{e^{x_{b}}\cdot e^{\frac{x-1}{x_{b}}}}{x_{b}\cdot (e^{x_{b}}-1)}$$

where *V* is the box volume, *n* is the total number of boxes, *x* is a non-dimensional variable describing the position along the flow path and varies from 0 at the high-temperature endmember to 1 at the point where fluid is venting into the deep ocean, and *x*_*b*_ is a shape parameter describing the geometry of the subsurface mixing zone. The shape parameter allowed the model to transition between two different mixing regimes. If *x*_*b*_ >>1, then the flow path resembled a linear crack or straight pipe. If *x*_*b*_ <<1, then the flow path spread out as it rose, approximating an expanding plume percolating through the ocean crust. The fluid flux, *Q′*_vt_, and the shape parameter, *x*_*b*_, were treated as tuning parameters that were adjusted to understand how flow characteristics influence the chemical concentrations and the microbial populations present in venting fluids. While *Q′*_vt_ sets the timescale of fluid flow, the shape parameter, *x*_*b*_, determined the relative amount of time spent at various temperatures along the flow path. All model parameters and boundary conditions are provided in Table S5.

The model state variables were concentration of CH_4_, [CH_4_] (μmol/kg), concentration of H_2_, [H_2_] (μmol/kg), concentration of a thermophilic methanogen with *M. thermolithotrophicus* growth kinetics, [*M_the_*] (cells/L), concentration of a hyperthermophilic methanogen with *M. jannaschii* growth kinetics, [*M_jan_*] (cells/L), concentration of a thermophilic thiosulfate reducer with *D. thermolithotrophum* growth kinetics, [*D_the_*] (cells/L), concentration of Mg^2+^, [Mg^2+^] (mmol/kg), and temperature, *T* (K). Temperature was calculated assuming pure mixing and was given by the following equation,


(Eqn. 2) $$T=\frac{f_{ht}C_{p,ht}T_{ht}+\left(1-f_{ht}\right)C_{p,sw}T_{sw}}{f_{ht}C_{p,ht}+\left(1-f_{ht}\right)C_{p,sw}}$$

where *f*_ht_ is the fraction of high-temperature fluid in the box determined from the Mg^2+^ content of the venting fluid assuming zero Mg^2+^ in the hydrothermal endmember, *T*_ht_ is the temperature of the hydrothermal endmember fluid, *T*_sw_ is the temperature of seawater, and *C*_*p*,ht_ and *C*_*p*,sw_ are the heat capacities of hydrothermal endmember fluid and seawater, respectively. During hydrothermal circulation, Mg^2+^ is removed from solution within hours at high temperatures, and the Mg^2+^ content of diffuse fluid indicates how much hot, zero-Mg^2+^ endmember is in the fluid (32).

The rest of the variables were described by the following system of differential equations for each box *i*, which were solved using the method-of-lines. The model was implemented in the R software environment using the package ReacTran (33).


(Eqn. 3) $$\frac{d{\left[H_{2}\right]}_{i}}{dt}=-\frac{\Delta_{i}\left(Q\cdot \left[H_{2}\right]\right)}{\Delta V_{i}}+\frac{Q_{sw}\cdot {\left[H_{2}\right]}^{sw}}{\Delta V_{i}}-4\sum_{i=1}^{2}R_{CH4_{i,j}}-4R_{H2S_{i,j}}$$

(Eqn. 4) $$\frac{{{d[CH}_{4}]}_{i}}{dt}=-\frac{{∆}_{i}\left(Q∙\left[{CH}_{4}\right]\right)}{{∆V}_{i}}+\frac{Q_{sw}∙{\left[{CH}_{4}\right]}^{sw}}{{∆V}_{i}}+\sum_{i=1}^{2}R_{{CH4}_{i,j}}$$

(Eqn. 5) $$\frac{{d[M_{the}]}_{i}}{dt}=-\frac{{∆}_{i}\left(Q∙\left[M_{the}\right]\right)}{{∆V}_{i}}+R_{M_{the-i}}$$

(Eqn. 6) $$\frac{{d[M_{jan}]}_{i}}{dt}=-\frac{{∆}_{i}\left(Q∙\left[M_{jan}\right]\right)}{{∆V}_{i}}+R_{M_{jan-i}}$$

(Eqn. 7) $$\frac{{d[D_{the}]}_{i}}{dt}=-\frac{{∆}_{i}\left(Q∙\left[D_{the}\right]\right)}{{∆V}_{i}}+R_{S_{i}}$$

(Eqn. 8) $$\frac{{d[Mg]}_{i}}{dt}=-\frac{{∆}_{i}(Q∙\left[Mg\right])}{∆V_{i}}+\frac{Q_{sw}{∙[Mg]}^{sw}}{{∆V}_{i}}$$

where *Q* is fluid flux. Methane (*R*_CH4_) and sulfide (*R*_H2S_) production are calculated by,


(Eqn. 9) $$R_{CH4}=10^{-9}∙v_{max}∙\frac{\left[H_{2}\right]}{\left[H_{2}\right]+K_{H_{2}}}∙\frac{e^{\left(T_{max}-T\right)}}{1+e^{\left(T_{max}-T\right)}}$$

(Eqn. 10) $$R_{H2S}=\frac{\mu_{max}}{0.693∙Y_{x/p}}∙\frac{\left[H_{2}\right]}{\left[H_{2}\right]+K_{H2}}∙\frac{e^{\left(T_{max}-T\right)}}{1+e^{\left(T_{max}-T\right)}}$$

where, for CH_4_ production, *v*_max_ is the maximum rate of cell-specific CH_4_ production, *K*_H2_ is the half-saturation constant for cell-specific CH_4_ production, and *T*_max_ is the optimum growth temperature of the methanogen or sulfur reducer (12). A conversion factor of 10^−9^ is used to convert *v*_max_, which in Table S5 is expressed in terms of fmol/cell/h to μmol/cell/h. For H_2_S production, *Y*_*x*/*p*_ is the growth yield (this study) and μ_max_ is the maximum growth rate (17).

The growth rates for methanogens (*R*_*M*_) and thiosulfate reducers (*R*_*S*_) are given by,


(Eqn. 11) $$R_{M}=Ae^{-\frac{E_{a}}{R_{g}T}}\cdot \left[M_{the\;or\;jan}\right]\cdot \frac{\left[H_{2}\right]}{\left[H_{2}\right]+K_{H2}}\cdot \frac{e^{\left(T_{max}-T\right)}}{1+e^{\left(T_{max}-T\right)}}$$

(Eqn. 12) $$R_{S}={Ae}^{-\frac{E_{a}}{R_{g}T}}∙[D_{the}]∙\frac{\left[H_{2}\right]}{\left[H_{2}\right]+K_{H2}}∙\frac{e^{\left(T_{max}-T\right)}}{1+e^{\left(T_{max}-T\right)}}$$

where *A* is the Arrhenius constant, *E*_*a*_ is the activation energy. These parameters were drawn from Stewart et al. (12, 17).

### Field constraints on the reactive transport model

Field measurements of *Methanocaldococcus* species, *Methanothermococcus* species, *Desulfurobacterium* species, CH_4_, and H_2_ concentrations in exiting fluids at individual diffuse vents were used to constrain the modeled subseafloor methanogen and sulfur reducer abundances, CH_4_ production, and the shape function of fluid mixing for each vent as in Stewart et al. (12). The top five best fits of the model to field data were determined by calculating the sum of squared variable costs using the modCost function from the R package FEM (34). Dissolved inorganic carbon was also measured in the fluids to ensure that it was not growth limiting. Thirty-seven diffuse hydrothermal fluid samples were collected from Marker 113 and Marker 33 in 2013, 2014, and 2015 using the deep-sea remotely operated vehicles *Jason* II and *ROPOS* (Table S1) as previously described (4, 5). The concentration of *Methanocaldococcus*, *Methanothermococcus*, and *Desulfurobacterium* cells in the diffuse hydrothermal fluids was estimated from the product of the proportion of these organisms in annotated metagenome sequence read counts (4) and the total cell counts (5) (Table S1). Functionally annotated reads from metagenomes collected in 2013, 2014, and 2015 from Marker 113 and Marker 33 were taxonomically classified and proportioned relative to the whole metagenome for each sample (4). The proportions of annotated reads for *Methanothermococcus*, *Methanocaldococcus*, and *Desulfurobacterium* were used to estimate their concentrations relative to the total number of cells in the same samples.

There was no high-temperature hydrothermal venting within 0.5 km of Marker 113 or Marker 33 that could provide hydrothermal endmember H_2_ concentrations. Therefore, high-temperature endmember H_2_ concentrations for these sites were estimated based on a trend of endmember H_2_ and endmember Cl^−^ for the closest high-temperature vents at Axial Seamount (12). The endmember Cl^−^ concentration of Marker 113 is ~100 mmol/kg and that of Marker 33 is ~400 mmol/kg (from extrapolation of diffuse fluid Cl^−^ to zero Mg^2+^). Temperature and chlorinity for the high-temperature endmembers were determined from the relationship of Mg^2+^ and temperature (or chlorinity) and extrapolated to zero Mg^2+^ concentration. The nearest high-temperature vents with similar Cl^−^ endmembers are Diva vent (endmember Cl^−^ 200 mmol/kg, endmember H_2_ 400–970 μmol/kg) and El Guapo vent (endmember Cl^−^ 400 mmol/kg and endmember H_2_ 120–470 μmol/kg). Endmember H_2_ concentrations at Marker 113 and Marker 33 were assigned values of 950 and 300 µmol/kg, respectively (12). These endmember H_2_ values for the diffuse vent sites reflect the fact that Marker 113 has a vapor-dominated source with higher gas content than the source for Marker 33.

## EDITOR'S NOTE

This article was handled by Betül Kaçar, who acted as a Guest Editor in consultation with the Editor in Chief, Gemma Reguera.

## SUPPLEMENTAL MATERIAL

Supplemental figures and tables aem.00292-24-s0001.docx Fig. S1-S5; Tables S1-S5.

ASM does not own the copyrights to Supplemental Material that may be linked to, or accessed through, an article. The authors have granted ASM a non-exclusive, world-wide license to publish the Supplemental Material files. Please contact the corresponding author directly for reuse.
