# Supplementary material for: Non-thermodynamic factors affect competition between thermophilic chemolithoautotrophs from deep-sea hydrothermal vents
Source: Appl Environ Microbiol. 2024 Jul 16;90(8):e00292-24. doi: 10.1128/aem.00292-24 (PMC11337833; doi:10.1128/aem.00292-24)
Supplement: Supplemental figures and tables — Fig. S1-S5; Tables S1-S5. [file aem.00292-24-s0001.docx]

**Supplemental Material for**

**Non-Thermodynamic Factors Affect Competition Between Thermophilic Chemolithoautotrophs from Deep-Sea Hydrothermal Vents**

Briana C. Kubik,^a^ James F. Holden,^a^#

*^a^Department of Microbiology, University of Massachusetts, Amherst, Massachusetts, USA*

#Address correspondence to James F. Holden, jholden@umass.edu.

This file includes:

Figures S1 through S5

Tables S1 through S5


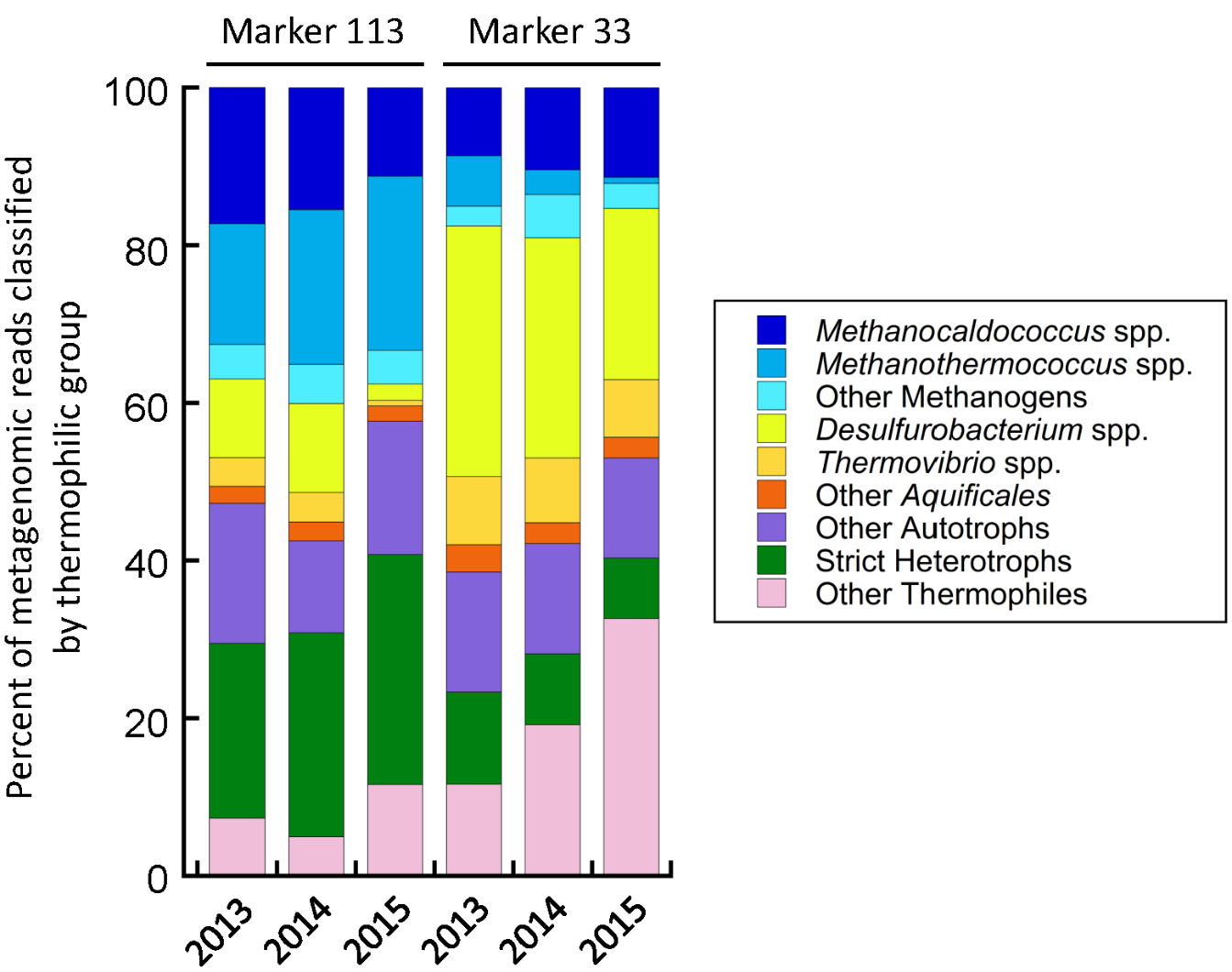


FIG S1. Taxonomic classification of functionally annotated reads from metagenomes of thermophiles that grow optimally above 65°C (after reference 8). Relative abundance of major taxonomic and functional groups of organisms are shown. Other Methanogens consists of *Methanotorris*, *Methanopyrus*, *Methanothermus*, and *Methanothermobacter* species; Other Aquificales consists of *Aquifex, Balnearium, Caldimicrobium, Hydrogenivirga, Hydrogenobacter, Persephonella, Phorcysia, Sulfurihydrogenium, Thermocrinis, Thermosulfidibacter,* and *Venenivibrio* species; Other Autotrophs consists of *Geothermobacterium*, *Thermodesulfatator*, *Thermodesulfobacterium*, and *Thermosulfurimonas* species; Strict Heterotrophs consists of *Pyrococcus*, *Thermococcus*, *Thermotoga*, *Fervidobacterium*, *Caldicellulosiruptor*, and *Thermoanaerobacter* species; and Other Thermophiles consists of *Archaeoglobus*, *Ferroglobus*, *Geoglobus*, *Aeropyrum*, *Desulfurococcus*, *Hyperthermus*, *Ignicoccus*, *Ignisphaera*, *Pyrodictium*, *Pyrolobus*, *Staphylothermus*, *Stetteria*, *Sulfophobococcus*, *Thermodiscus*, *Thermogladius*, *Thermosphaera*, *Caldivirga*, *Pyrobaculum*, *Thermocladium*, *Thermofilum*, *Thermoproteus*, *Vulcanisaeta*, *Acidilobus*, *Caldisphaera*, *Acidianus*, *Metallosphaera*, *Stygioglobus*, *Sulfolobus*, and *Sulfurisphaera* species (as grouped by 1).


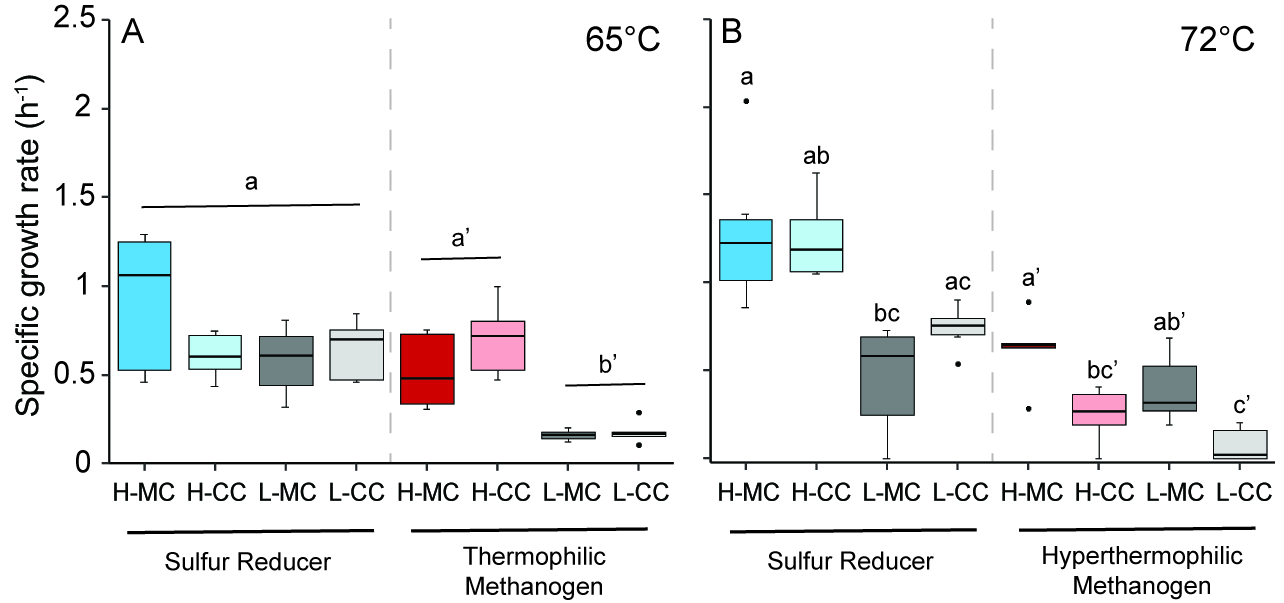


FIG S2. Specific growth rate of *D. thermolithotrophum* and *M. thermolithotrophicus* at 65°C (A) and *D. thermolithotrophum* and *M. jannaschii* at 72°C (B). *D. thermolithotrophum* at high initial H_2_ concentration is shown in blue shades, methanogens at high initial H_2_ concentration are shown in red shades, and *D. thermolithotrophum* and methanogens at low initial H_2_ concentrations are both shown in grey shades. Monocultures are the darker shades of all colors while cocultures are the lighter shades. The statistical relevance (*P* < 0.05) of the data is shown separately for each organism (non-prime versus prime). Abbreviations: MC, monoculture; CC, coculture in 1:1 initial cell ratio; H, high initial H_2_ concentration; L, low initial H_2_ concentration.


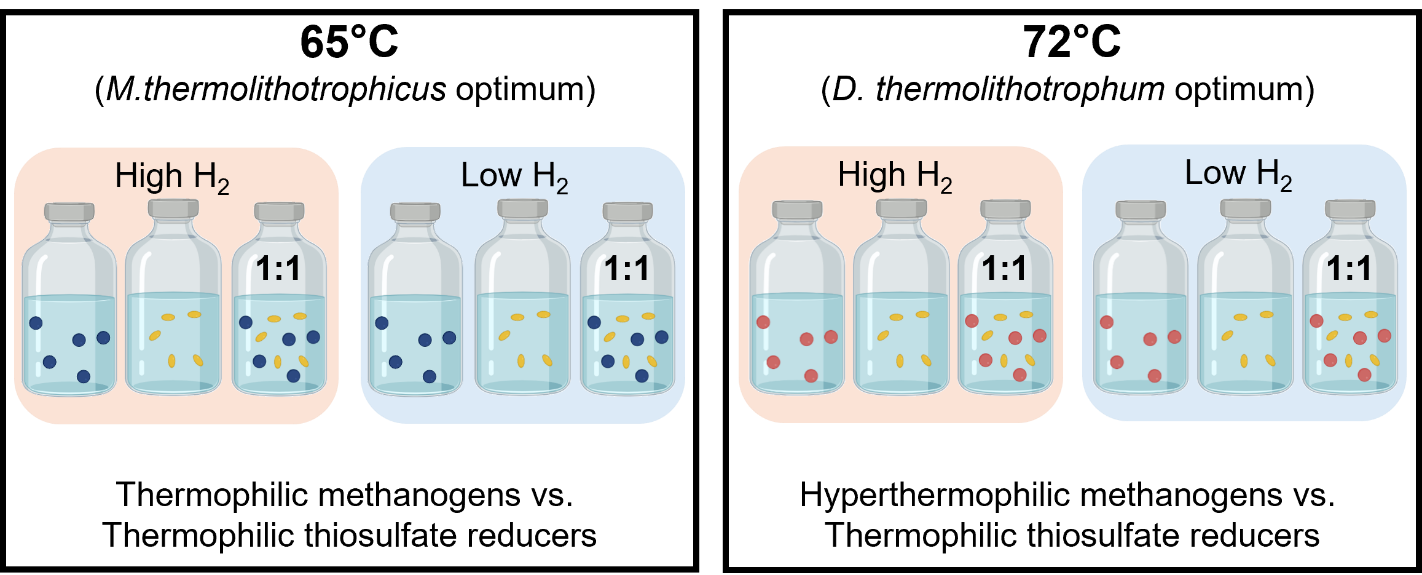


**FIG S3.** Experimental setup for the mono- and coculture experiments at high and low H_2_ concentrations. The methanogens (large red circle) and the thiosulfate reducer (short yellow rods) were each grown in monoculture and in methanogen-thiosulfate reducer coculture. All mono-and coculture media were inoculated with 10^6^ cells/ml. *M. thermolithotrophicus* and *D. thermolithotrophum* were incubated at 65°C while *M. jannaschii* and *D. thermolithotrophum* were incubated at 72°C. For each temperature, half of the bottles were incubated with 1.6 atm of H_2_ and 0.4 atm of CO_2_; the other half, 0.14 atm of H_2_, 1.46 atm of N_2_, and 0.4 atm of CO_2_. Created with BioRender.com.


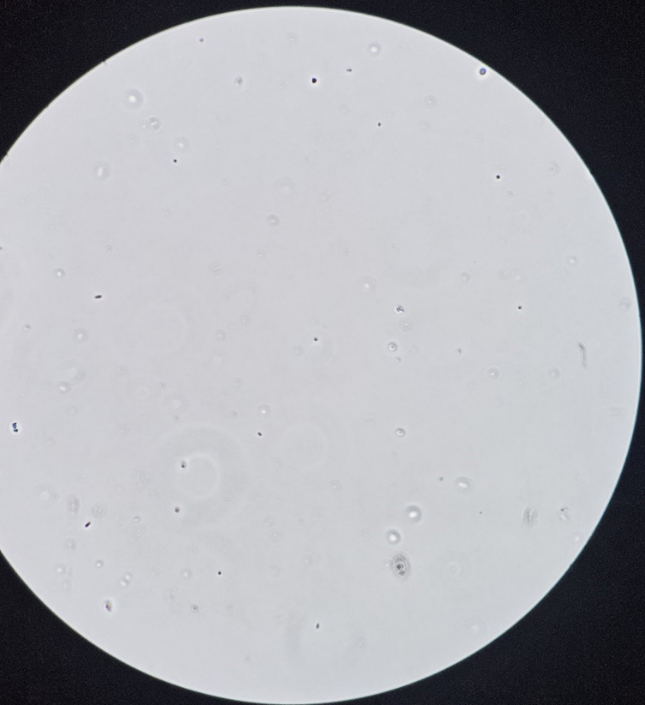

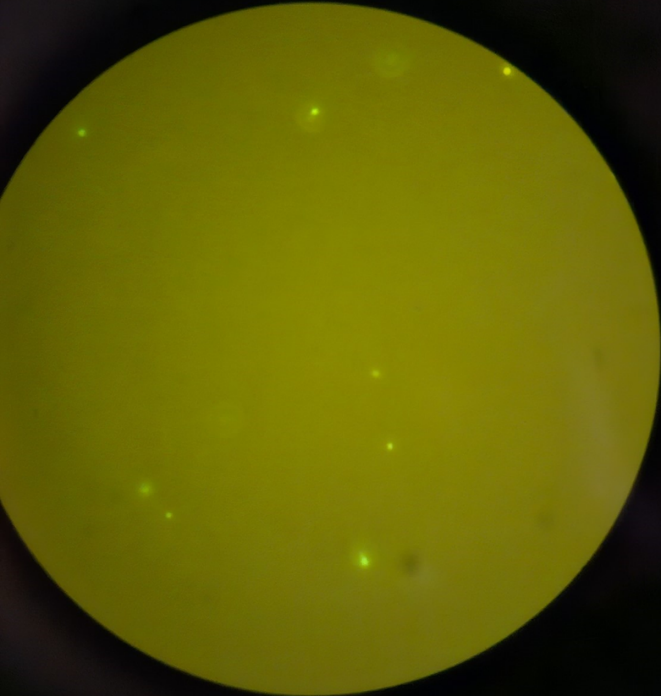


**FIG S4.** Phase contrast light (left) and F_420_ autofluoresence (right) microscopy of a coculture of *D. thermolithotrophum* and *M. thermolithotrophicus.*


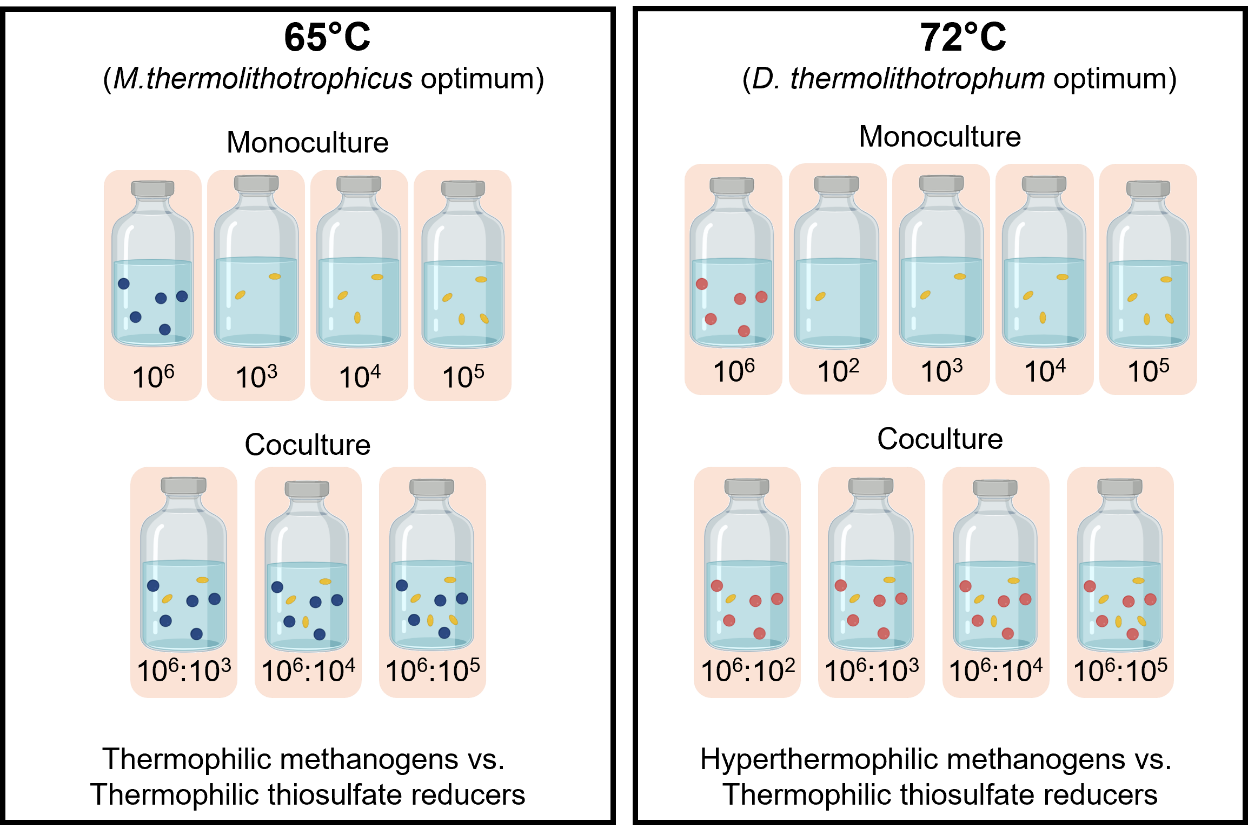


**FIG S5.** Experimental setup for the mono- and coculture experiments at varying proportions of methanogens and thiosulfate reducers. The methanogens (large red circle) and the thiosulfate reducer (short yellow rods) were each grown in monoculture and in methanogen-thiosulfate reducer coculture. The methanogen was always inoculated at 10^6^ cells/ml while *D. thermolithotrophum* was inoculated in mono- and coculture at 10^5^, 10^4^, 10^3^, and 10^2^ cells/ml. *M. thermolithotrophicus* and *D. thermolithotrophum* were incubated at 65°C while *M. jannaschii* and *D. thermolithotrophum* were incubated at 72°C. All the bottles were incubated with 1.6 atm of H_2_ and 0.4 atm of CO_2_. Created with Biorender.com.

Table S1. Fluid chemistry, total cell concentrations, and cell concentration estimates for hyperthermophilic (H) and thermophilic (T) methanogens and sulfur-reducing *Aquificae* based on their proportions of total cells for Marker 113 and Marker 33 vents (from 2, 3)

| Sample ID | T  (°C) | [Mg^2+^]  (mmol/kg) | [total cell]  (1/ml) | [methanogen]_H_  (1/ml) | [methanogen]_T_  (1/ml) | [S reducer]_T_  (1/ml) | [H_2_]  (µmol/kg) | [CH_4_]  (µmol/kg) | [CO_2_]  (mmol/kg) | [H_2_S]  (mmol/kg) |
| --- | --- | --- | --- | --- | --- | --- | --- | --- | --- | --- |
| **Mk. 113 (2013):** |  |  |  |  |  |  |  |  |  |  |
| Metagenome |  |  |  | 1.89% | 1.45% | 1.23% |  |  |  |  |
| R1662-IGT-02 | 21.1 | 51.12 | - | - | - | - | <1 | 17.732 | 4.5 | 0.216 |
| R1662-IGT-03 | 23.3 | 50.39 | - | - | - | - | <1 | 18.272 | 4.6 | 0.414 |
| R1663-HFS-01 | 24.1 | 50.34 | - | - | - | - | < 1 | 13.240 | - | 0.579 |
| R1663-HFS-02 | 24.0 | 49.82 | 2.63×10^5^ | 4.97×10^3^ | 3.81×10^3^ | 3.23×10^3^ | 1.350 | 14.400 | - | 0.582 |
| R1663-HFS-04 | 23.5 | 49.18 | 4.76×10^5^ | 9.00×10^3^ | 6.90×10^3^ | 5.85×10^3^ | < 1 | 13.050 | - | 0.576 |
| R1663-HFS-07 | 24.4 | 50.19 | 5.45×10^5^ | 1.03×10^4^ | 7.90×10^3^ | 6.70×10^3^ | < 1 | 16.333 | - | 0.664 |
| R1663-HFS-08 | 24.5 | 50.23 | 4.56×10^5^ | 8.62×10^3^ | 6.61×10^3^ | 5.61×10^3^ | < 1 | 16.867 | - | 0.729 |
| **Mk. 113 (2014):** |  |  |  |  |  |  |  |  |  |  |
| Metagenome |  |  |  | 2.46% | 2.47% | 1.85% |  |  |  |  |
| J786-HFS-01 | 24.3 | 50.49 | 6.80×10^5^ | 1.67×10^4^ | 1.68×10^4^ | 1.26×10^4^ | 0.004 | 34.836 | - | 0.556 |
| J786-HFS-03 | 25.1 | 50.46 | 8.50×10^5^ | 2.09×10^4^ | 2.10×10^4^ | 1.57×10^4^ | - | - | - | - |
| J786-HFS-08 | 24.9 | 50.42 | 2.30×10^6^ | 5.66×10^4^ | 5.68×10^4^ | 4.26×10^4^ | - | - | - | 0.516 |
| J786-IGT-11 | 26.4 | 50.37 | - | - | - | - | 1.700 | 36.700 | 4.8 | 0.711 |
| J786-IGT-12 | 27.4 | 50.35 | - | - | - | - | 0.600 | 40.300 | 7.4 | 0.685 |
| J791-HFS-06 | 31.1 | 49.89 | - | - | - | - | 0.722 | 49.324 | - | 0.697 |
| J791-IGT-07 | 27.4 | 49.97 | - | - | - | - | 1.100 | 43.300 | 5.5 | 0.024 |
| **Mk. 113 (2015):** |  |  |  |  |  |  |  |  |  |  |
| Metagenome |  |  |  | 1.34% | 2.08% | 0.25% |  |  |  |  |
| J824-HFS-13 | 25.4 | 49.72 | 2.50×10^6^ | 3.35×10^4^ | 5.20×10^4^ | 6.25×10^3^ | - | - | - | - |
| J824-HFS-14 | 25.2 | 49.72 | - | - | - | - | 0.390 | 17.443 | - | 0.484 |
| J824-HFS-18 | 25.4 | 50.17 | 1.50×10^6^ | 2.01×10^4^ | 3.12×10^4^ | 3.75×10^3^ | 0.240 | 22.270 | - | 0.578 |
|  |  |  |  |  |  |  |  |  |  |  |
| **Mk. 33 (2013):** |  |  |  |  |  |  |  |  |  |  |
| Metagenome |  |  |  | 1.14% | 0.76% | 4.37% |  |  |  |  |
| R1665-HFS-01 | 27.6 | 45.80 | - | - | - | - | 2.600 | 53.530 | - | 0.478 |
| R1665-HFS-02 | 28.3 | 45.55 | - | - | - | - | < 1 | 17.996 | - | 0.591 |
| R1665-HFS-03 | 27.8 | 44.97 | 2.95×10^5^ | 3.36×10^3^ | 2.24×10^3^ | 1.29×10^4^ | 1.231 | 15.824 | - | 0.561 |
| R1665-HFS-05 | 27.3 | 45.89 | 4.17×10^5^ | 4.75×10^3^ | 3.17×10^3^ | 1.82×10^4^ | < 1 | 18.987 | - | 0.545 |
| R1665-HFS-06 | 26.7 | 45.89 | 2.79×10^5^ | 3.18×10^3^ | 2.12×10^3^ | 1.22×10^4^ | 1.850 | 18.400 | - | 0.491 |
| R1665-IGT-08 | 39.4 | 42.96 | - | - | - | - | 1.310 | 30.122 | 15.1 | 0.734 |
| R1665-HFS-09 | 27.0 | 46.69 | - | - | - | - | < 1 | 10.850 | - | 0.397 |
|  |  |  |  |  |  |  |  |  |  |  |
|  |  |  |  |  |  |  |  |  |  |  |
|  |  |  |  |  |  |  |  |  |  |  |
|  |  |  |  |  |  |  |  |  |  |  |
|  |  |  |  |  |  |  |  |  |  |  |
| (*cont. next page*) |  |  |  |  |  |  |  |  |  |  |
|  |  |  |  |  |  |  |  |  |  |  |
| Sample ID | T  (°C) | [Mg^2+^]  (mmol/kg) | [total cell]  (1/ml) | [methanogen]_H_  (1/ml) | [methanogen]_T_  (1/ml) | [S reducer]_T_  (1/ml) | [H_2_]  (µmol/kg) | [CH_4_]  (µmol/kg) | [CO_2_]  (mmol/kg) | [H_2_S]  (mmol/kg) |
| **Mk. 33 (2014):** |  |  |  |  |  |  |  |  |  |  |
| Metagenome |  |  |  | 2.78% | 0.74% | 6.67% |  |  |  |  |
| J790-HFS-05 | 19.0 | 49.10 | 2.95×10^5^ | 8.20×10^3^ | 2.18×10^3^ | 1.97×10^4^ | - | - | - | - |
| J790-HFS-06 | 18.5 | 48.12 | 3.85×10^5^ | 1.07×10^4^ | 2.85×10^3^ | 2.57×10^4^ | 0.033 | 6.357 | - | 0.261 |
| J790-HFS-07 | 18.7 | 48.36 | 3.50×10^5^ | 9.73×10^3^ | 2.59×10^3^ | 2.33×10^4^ | 0 | 3.697 | - | 0.265 |
| J790-IGT-11 | 21.3 | 46.25 | - | - | - | - | 1.300 | 18.500 | 12.2 | 0.307 |
| J790-IGT-12 | 31.8 | 44.09 | - | - | - | - | 1.600 | 24.300 | 9.7 | 0.539 |
| **Mk. 33 (2015):** |  |  |  |  |  |  |  |  |  |  |
| Metagenome |  |  |  | 2.21% | 0.27% | 4.72% |  |  |  |  |
| J822-HFS-05 | 33.1 | 44.75 | 6.30×10^5^ | 1.39×10^4^ | 1.70×10^3^ | 2.97×10^4^ | - | - | - | 0.540 |
| J822-HFS-06 | 33.0 | 44.49 | - | - | - | - | 0.092 | 19.215 | - | - |
| J822-HFS-07 | 34.0 | 44.90 | 8.10×10^5^ | 1.79×10^4^ | 2.19×10^3^ | 3.82×10^4^ | 0.136 | 19.721 | - | 0.639 |
| J825-HFS-05 | 40.6 | 44.50 | - | - | - | - | 0.132 | 25.700 | - | 0.628 |
| J825-HFS-06 | 40.5 | 43.03 | 1.57×10^5^ | 3.47×10^3^ | 424 | 7.41×10^3^ | - | - | - | 0.264 |
| J825-HFS-07 | 40.3 | 42.78 | 3.60×10^5^ | 7.96×10^3^ | 972 | 1.70×10^4^ | - | - | - | 0.731 |

**Table S2.** Maximum cell concentration, specific growth rate, and total product (CH_4_ or H_2_S/bottle) data for *M. jannaschii*, *M. thermolithotrophicus*, and *D. thermolithotrophum* mono- and cocultures at 72°C and 65°C with high (1.2 mM) or low (85 µM) H_2_

| **Sample** | **High H_2_**  **monoculture** | | | **High H_2_**  **coculture** | | | **Low H_2_**  **monoculture** | | | **Low H_2_**  **coculture** | | |
| --- | --- | --- | --- | --- | --- | --- | --- | --- | --- | --- | --- | --- |
|  | **[Cell]_max_**  (cells/mL) | **Specific growth rate**  (h^-1^) | **Total product**  (µmol) | **[Cell]_max_**  (cells/mL) | **Specific growth rate**  (h^-1^) | **Total product**  (µmol) | **[Cell]_max_**  (cells/mL) | **Specific growth rate**  (h^-1^) | **Total product**  (µmol) | **[Cell]_max_**  (cells/mL) | **Specific growth rate**  (h^-1^) | **Total product**  (µmol) |
| ***M. jannaschii* at 72°C:** | | |  |  |  |  |  |  |  |  |  |  |
| 1 | 7.25×10^6^ | 0.2821 | 42.1 | 4.04×10^6^ | 0.2337 | 10.5 | 2.25×10^6^ | 0.6864 | 8.72 | 7.43×10^5^ | 0 | 1.25 |
| 2 | 1.08×10^7^ | 0.635 | 65.72 | 3.99×10^6^ | 0.1761 | 26.55 | 1.70×10^6^ | 0.522 | 10.54 | 1.32×10^6^ | 0.036 | 1.95 |
| 3 | 9.60×10^6^ | 0.6262 | 93.22 | 2.02×10^6^ | 0.2967 | 15.73 | 1.95×10^6^ | 0.1903 | 5.89 | 7.90×10^5^ | 0.0024 | 1.5 |
| 4 | 7.30×10^6^ | 0.652 | 84.75 | 1.74×10^6^ | 0 | 24.06 | 2.15×10^6^ | 0.2675 | 10.51 | 4.67×10^5^ | 0 | 0.26 |
| 5 | 9.30×10^6^ | 0.8921 | 141.6 | 6.79×10^6^ | 0.3871 | 22.75 | 2.25×10^6^ | 0.3152 | 7.34 | 1.35×10^6^ | 0.1991 | 2.33 |
| 6 | 8.75×10^6^ | 0.6481 | 175.22 | 4.99×10^6^ | 0.4059 | 8.94 | - | - | - | 1.15×10^6^ | 0.1957 | 2.7 |
| ***D. thermolithotrophum* at 72°C:** | | |  |  |  |  |  |  |  |  |  |  |
| 1 | 2.52×10^7^ | 0.957 | 279.46 | 1.45×10^7^ | 1.0552 | 230.5 | 3.82×10^7^ | 0.1371 | 0 | 1.60×10^7^ | 0.5376 | 0 |
| 2 | 3.27×10^7^ | 0.8575 | 277.59 | 1.36×10^7^ | 1.0745 | 214.73 | 3.47×10^7^ | 0 | 0 | 2.07×10^7^ | 0.6921 | 0 |
| 3 | 1.81×10^7^ | 1.2667 | 345.08 | 1.52×10^7^ | 1.3821 | 320.7 | 2.83×10^7^ | 0.5651 | 0 | 2.11×10^7^ | 0.812 | 0 |
| 4 | 2.90×10^7^ | 1.1839 | 318.44 | 1.26×10^7^ | 1.0491 | 335.86 | 2.95×10^7^ | 0.5952 | 0 | 2.81×10^7^ | 0.8989 | 0 |
| 5 | 2.61×10^7^ | 2.033 | 357.76 | 1.78×10^7^ | 1.6262 | 335.62 | 2.31×10^7^ | 0.7249 | 0 | 2.17×10^7^ | 0.745 | 0 |
| 6 | 2.31×10^7^ | 1.3898 | 358.22 | 2.16×10^7^ | 1.2929 | 228.08 | 2.13×10^7^ | 0.7298 | 0 | 2.89×10^7^ | 0.7578 | 0 |
| ***M. thermolithotrophicus* at 65°C:** | | |  |  |  |  |  |  |  |  |  |  |
| 1 | 1.44×10^7^ | 0.3395 | 126.93 | 3.07×10^6^ | 0.7191 | 66.43 | 3.40×10^6^ | 0.16 | 8.49 | 2.39×10^6^ | 0.1057 | 5.69 |
| 2 | 1.36×10^7^ | 0.3335 | 127.89 | 2.47×10^6^ | 0.9994 | 6.42 | 5.75×10^6^ | 0.1412 | 27.1 | 3.00×10^6^ | 0.1817 | 6.93 |
| 3 | 1.01×10^7^ | 0.307 | 104.27 | 6.86×10^5^ | 0.5097 | 78.88 | 9.50×10^5^ | 0.1263 | 5.93 | 1.25×10^6^ | 0.2923 | 0.78 |
| 4 | 5.95×10^6^ | 0.7577 | 82.07 | 1.49×10^6^ | 0.473 | 30.5 | 1.10×10^6^ | 0.1778 | 4.2 | 7.50×10^5^ | 0.1544 | 1.27 |
| 5 | 5.80×10^6^ | 0.7085 | 103.26 | 1.83×10^6^ | 0.5437 | 41.41 | 9.00×10^5^ | 0.2062 | 0 | 7.50×10^5^ | 0.1709 | 1.7 |
| 6 | 6.20×10^6^ | 0.483 | 90.51 | 2.50×10^6^ | 0.7231 | 60.63 | - | - | - | - | - | - |
| 7 | 6.55×10^6^ | 0.7514 | 103.51 | 2.90×10^6^ | 0.8829 | 75.57 | - | - | - | - | - | - |
| ***D. thermolithotrophum* at 65°C:** | | |  |  |  |  |  |  |  |  |  |  |
| 1 | 2.06×10^7^ | 0.459 | 172.47 | 4.43×10^6^ | 0.4361 | 100.28 | 6.55×10^6^ | 0.3238 | 0 | 2.50×10^7^ | 0.4592 | 0 |
| 2 | 2.40×10^7^ | 0.5217 | 228.37 | 8.58×10^6^ | 0.5053 | 96.35 | 2.30×10^7^ | 0.3992 | 0 | 2.03×10^7^ | 0.4748 | 0 |
| 3 | 2.52×10^7^ | 0.5307 | 249.44 | 8.11×10^6^ | 0.5619 | 72.75 | 2.75×10^7^ | 0.7484 | 0 | 2.20×10^7^ | 0.7567 | 0 |
| 4 | 1.31×10^7^ | 1.2513 | 170.44 | 3.7×10^6^ | 0.7514 | 110.04 | 3.15×10^7^ | 0.5745 | 0 | 2.18×10^7^ | 0.8459 | 0 |
| 5 | 1.70×10^7^ | 1.2965 | 143.61 | 4.37×10^6^ | 0.7196 | 122.81 | 1.75×10^7^ | 0.8078 | 0 | 1.93×10^7^ | 0.6993 | 0 |
| 6 | 1.24×10^7^ | 1.254 | 174.64 | 6.70×10^6^ | 0.726 | 127.89 | 3.00×10^7^ | 0.6393 | 0 | - | - | - |
| 7 | 1.84×10^7^ | 1.0146 | 181.05 | 7.80×10^6^ | 0.6042 | 51.14 | - | - | - | - | - | - |
| 8 | 1.84×10^7^ | 1.1018 | 236.99 | - | - | - | - | - | - | - | - | - |

| **Sample** | **Coculture 10:1** | | **Coculture 10^2^:1** | | **Coculture 10^3^:1** | | **Coculture 10^4^:1** | |
| --- | --- | --- | --- | --- | --- | --- | --- | --- |
|  | **[Cell]_max_**  (cells/mL) | **Total product**  (µmol) | **[Cell]_max_**  (cells/mL) | **Total product**  (µmol) | **[Cell]_max_**  (cells/mL) | **Total product**  (µmol) | **[Cell]_max_**  (cells/mL) | **Total product**  (µmol) |
| ***M. jannaschii* at 72°C:** | | |  |  |  |  |  |  |
| 1 | 2.80×10^7^ | 396.29 | 1.28×10^7^ | 448.15 | 2.08×10^7^ | 409.21 | 2.96×10^7^ | 428.52 |
| 2 | 1.76×10^7^ | 410.02 | 4.37×10^7^ | 369.3 | 2.64×10^7^ | 357.14 | 1.84×10^7^ | 463.89 |
| 3 | 2.56×10^7^ | 457.49 | 1.67×10^7^ | 451.67 | 2.32×10^7^ | - | 2.64×10^7^ | 442.73 |
| 4 | 3.04×10^7^ | - | 2.56×10^7^ | - | - | - | 3.20×10^7^ | 437.84 |
| ***D. thermolithotrophum* at 72°C:** | | |  |  |  |  |  |  |
| 1 | 1.86×10^8^ | 299.82 | 1.42×10^8^ | 259.82 | 2.06×10^8^ | 306.91 | 1.30×10^8^ | 297.09 |
| 2 | 1.34×10^8^ | 292.36 | 1.83×10^8^ | 274.18 | 1.78×10^8^ | 306.36 | 1.26×10^8^ | 277.27 |
| 3 | 1.82×10^8^ | 301.09 | 1.23×10^8^ | 300 | 9.44×10^7^ | 329.64 | 2.44×10^8^ | 323.09 |
| 4 | 9.76×10^7^ | 310.91 | 2.64×10^8^ | 300 | - | - | 2.22×10^8^ | 326 |
| ***M. thermolithotrophicus* at 65°C:** | | |  |  |  |  |  |  |
| 1 | 3.04×10^7^ | 134.19 | 1.76×10^7^ | 60.86 | 8.50×10^7^ | 579.38 | - | - |
| 2 | 3.28×10^7^ | 472.23 | 2.40×10^7^ | 163.3 | 6.33×10^7^ | 617.28 | - | - |
| 3 | 7.11×10^7^ | 514.33 | 3.60×10^7^ | 364.49 | 1.19×10^8^ | 598.71 | - | - |
| 4 | 6.96×10^7^ | 485.55 | 5.36×10^7^ | 286.34 | 1.50×10^8^ | 602.87 | - | - |
| 5 | - | - | 2.64×10^7^ | - | - | - | - | - |
| ***D. thermolithotrophum* at 65°C:** | | |  |  |  |  |  |  |
| 1 | 2.08×10^8^ | 276.73 | 1.47×10^8^ | 306.55 | 1.80×10^7^ | 40.73 | - | - |
| 2 | 1.42×10^8^ | 148.55 | 1.30×10^8^ | 319.09 | 1.78×10^7^ | 16.36 | - | - |
| 3 | 2.31×10^8^ | 207.55 | 1.49×10^8^ | 281.27 | 2.32×10^7^ | 22.24 | - | - |
| 4 | 2.46×10^8^ | 269.39 | 8.88×10^7^ | 302.91 | 3.52×10^7^ | 13.06 | - | - |
| 5 | - | - | 1.45×10^8^ | - | - | - | - | - |

**Table S3.** Maximum cell concentration and total product (CH_4_ or H_2_S/bottle) data for *M. jannaschii*, *M. thermolithotrophicus*, and *D. thermolithotrophum* cocultures at 65°C and 72°C with methanogen:thiosulfate reducer ratios ranging from 10:1 to 10^4^:1

**Table S4.** Maximum cell concentration and total product (CH_4_ or H_2_S/bottle) data for *M. jannaschii*, *M. thermolithotrophicus*, and *D. thermolithotrophum* monocultures at 65°C and 72°C with initial cell concentrations ranging from 10^6^ cells/ml to 10^2^ cells/ml

| **Sample** | **Monoculture 10^6^ cells/ml** | | **Monoculture 10^5^ cells/ml** | | **Monoculture 10^4^ cells/ml** | | **Monoculture 10^3^ cells/ml** | | **Monoculture 10^2^ cells/ml** | |
| --- | --- | --- | --- | --- | --- | --- | --- | --- | --- | --- |
|  | **[Cell]_max_**  (cells/mL) | **Total product**  (µmol) | **[Cell]_max_**  (cells/mL) | **Total product**  (µmol) | **[Cell]_max_**  (cells/mL) | **Total product**  (µmol) | **[Cell]_max_**  (cells/mL) | **Total product**  (µmol) | **[Cell]_max_**  (cells/mL) | **Total product**  (µmol) |
| ***M. jannaschii* at 72°C:** | | |  |  |  |  |  |  |  |  |
| 1 | 7.00×10^7^ | 470.5828 | - | - | - | - | - | - | - | - |
| 2 | 1.11×10^8^ | 633.9604 | - | - | - | - | - | - | - | - |
| 3 | 7.20×10^7^ | 611.1645 | - | - | - | - | - | - | - | - |
| 4 | 5.50×10^7^ | - | - | - | - | - | - | - | - | - |
| ***D. thermolithotrophum* at 72°C:** | | |  |  |  |  |  |  |  |  |
| 1 | - | - | 1.80×10^8^ | 324 | 1.60×10^8^ | 317.64 | 1.32×10^8^ | 286.91 | 1.70×10^8^ | 328.91 |
| 2 | - | - | 1.32×10^8^ | 308.18 | 1.60×10^8^ | 322.91 | 1.50×10^8^ | 317.27 | 1.32×10^8^ | 278.91 |
| 3 | - | - | 1.70×10^8^ | 317.09 | 1.53×10^8^ | 297.64 | 1.02×10^8^ | 289.82 | 1.22×10^8^ | 366.73 |
| 4 | - | - | 1.34×10^8^ | 264 | 1.64×10^8^ | 321.82 | 1.40×10^8^ | 313.82 | 1.32×10^8^ | 346.94 |
| ***M. thermolithotrophicus* at 65°C:** | | |  |  |  |  |  |  |  |  |
| 1 | 2.36×10^8^ | 227.9302 | - | - | - | - | - | - | - | - |
| 2 | 9.10×10^7^ | 578.267 | - | - | - | - | - | - | - | - |
| 3 | 5.28×10^7^ | 590.8312 | - | - | - | - | - | - | - | - |
| 4 | 1.50×10^8^ | 608.9701 | - | - | - | - | - | - | - | - |
| ***D. thermolithotrophum* at 65°C:** | | |  |  |  |  |  |  |  |  |
| 1 | - | - | 1.70×10^8^ | 324.55 | 1.22×10^8^ | 309.09 | 1.55×10^8^ | 328.91 | - | - |
| 2 | - | - | 1.49×10^8^ | 330 | 2.31×10^8^ | 312.73 | 1.46×10^8^ | 278.91 | - | - |
| 3 | - | - | 1.47×10^8^ | 341.84 | 2.05×10^8^ | 348.98 | 1.78×10^8^ | 366.73 | - | - |
| 4 | - | - | 1.75×10^8^ | 365.92 | 1.40×10^8^ | 351.22 | 1.56×10^8^ | 346.94 | - | - |

Table S5. Parameters, values, and boundary conditions used for simulations.

| Parameter | Mkr 113 | Mkr 33 | Units | Reference |
| --- | --- | --- | --- | --- |
| *Transport parameters* | | | | |
| Heat capacity of seawater, C*_p,sw_* | 4.143 | 4.143 | kJ/kg/°C | 3 |
| Heat capacity of HT end member, *C_p,ht_* | 6.70 | 4.47 | kJ/kg | 3 |
| *Growth kinetics for M. jannaschii* | | | | |
| Arrhenius constant, *A* | 9.12x10^12^ | 9.12x10^12^ | 1/h | 3 |
| Activation energy, *E_a_* | 86.3 | 86.3 | kJ | 3 |
| H_2_ half saturation, *k_H2_* | 37 | 37 | μmol/L | 3 |
| Maximum growth temperature, *T_max_* | 85 | 85 | °C | 3 |
| Cell specific CH_4_ production, *ν*_max_ | 43 | 43 | fmol/cell/h | 3 |
| *Growth kinetics for M. thermolithotrophicus* | | | | |
| Arrhenius constant, *A* | 3.36x10^11^ | 3.36x10^11^ | 1/h | 3 |
| Activation energy, *E_a_* | 73.8 | 73.8 | kJ | 3 |
| H_2_ half saturation, *k_H2_* | 27 | 27 | μmol/L | 3 |
| Maximum growth temperature, *T_max_* | 70 | 70 | °C | 3 |
| Cell specific CH_4_ production, *ν*_max_ | 24 | 24 | fmol/cell/h | 3 |
| *Growth kinetics for D. thermolithotrophum* | | | | |
| Arrhenius constant, *A* | 9x10^12^ | 9x10^12^ | 1/h | 6 |
| Activation energy, *E_a_* | 83.55 | 83.55 | kJ | 6 |
| H_2_ half saturation, *k_H2_* | 30 | 30 | μmol/L | 6 |
| Maximum growth temperature, *T_max_* | 77 | 77 | °C | 6 |
| Cell specific growth rate, μ_max_ | 2.1687 | 2.1687 | 1/h | 6 |
| Cell yield, *Y*_x/p_ | 1.10x10^7^ | 1.10x10^7^ | cells/µmol H_2_S | this study |
| *Boundary conditions* | | | | |
| H_2_ in HT end member, H_2,ht_ | 950 | 300 | μmol/kg | 3 |
| H_2_ in seawater, H_2,sw_ | 0 | 0 | μmol/kg | 3 |
| CH_4_ in HT end member | 56 | 70 | μmol/kg | 3 |
| CH_4_ in seawater | 0 | 0 | μmol/kg | 3 |
| Temperature of HT end member, *T_ht_* | 330 | 214 | °C | 3 |
| Mg in HT end member, Mg*_ht_* | 0 | 0 | mmol/kg | 3 |
| Mg in seawater, Mg*_sw_* | 52.7 | 52.7 | mmol/kg | 3 |
| *M. jannaschii* in seawater | 0 | 0 | cells/L |  |
| *M. jannaschii* in HT end member | 1 | 1 | cells/L |  |
| *M. thermolithotrophicus* in seawater | 0 | 0 | cells/L |  |
| *M. thermolithotrophicus* in HT end member | 1-1,000 | 1-1,000 | cells/L |  |
| *D. thermolithotrophum* in seawater | 0 | 0 | cells/L |  |
| *D. thermolithotrophum* in HT end member | 1 | 1 | cells/L |  |

^*^Heat capacities are calculated using supcrt92 (4) implemented in the R package CHNOSZ (5).

**Supplemental Material References**

1. Topçuoğlu BD, Holden JF. 2019. Extremophiles: hot environments, p 263-269. *In* Schmidt TM (ed), Encyclopedia of microbiology, 4^th^ ed, vol 2. Elsevier, UK.
2. Topçuoğlu BD, Stewart LC, Morrison HG, Butterfield DA, Huber JA, Holden JF. 2016. Hydrogen limitation and syntrophic growth among natural assemblages of thermophilic methanogens at deep-sea hydrothermal vents. Front Microbiol 7:1240.
3. Fortunato CS, Larson B, Butterfield DA, Huber JA. 2018. Spatially distinct, temporally stable microbial populations mediate biogeochemical cycling at and below the seafloor in hydrothermal vent fluids. Environ Microbiol 20:769-784.
4. Johnson JW, Oelkers EH, Helgeson HC. 1992. SUPCRT92: a software package for calculating the standard molal thermodynamic properties of minerals, gases, aqueous species, and reactions from 1 to 5000 bar and 0 to 1000°C. Comput Geosci 18:899-947.
5. Dick JM. 2008. Calculation of the relative metastabilities of proteins using the CHNOSZ software package. Geochem Trans 9:10.
